# A93 A RETROSPECTIVE REVIEW OF FECAL DIVERSION SYSTEM USE IN A TERTIARY CARE INTENSIVE CARE UNIT

**DOI:** 10.1093/jcag/gwae059.093

**Published:** 2025-02-10

**Authors:** A Monod, Z Lin, A Rostom

**Affiliations:** University of Ottawa, Ottawa, ON, Canada; The Ottawa Hospital Research Institute, Ottawa, ON, Canada; University of Ottawa, Ottawa, ON, Canada

## Abstract

**Background:**

Fecal diversion systems (FDS) are frequently deployed in intensive care units (ICUs), although recommendations for their use are heterogeneous. Complications can include pain, bleeding, perforation, infection and death. Risk assessment prior to FDS use may reduce the incidence of complications.

**Aims:**

We analyzed FDS usage in ICUs at The Ottawa Hospital (TOH), to determine complication rates and identify patients who may be at greatest risk.

**Methods:**

We performed a retrospective chart review of FDS deployments in TOH ICUs, from July 2021-July 2023. Continuous variables were analyzed for mean and standard deviation, and significance was determined using unpaired t-tests. Categorical variables were recorded as counts and percentages, and significance determined using chi-squared tests.

**Results:**

263 patients were included. 156 (59.3%) were male, and the mean age was 59.7. The most common admission diagnoses were sepsis (10.3%), COVID (7.6%), and respiratory failure (7.3%). The mean FDS duration was 11.6 days. FDS were most frequently deployed for diarrhea (85.0%), and perineal wounds (9.3%). Further patient characteristics are summarized in Table 1. 25(9.5%) patients developed new clinical bleeding after FDS placement, after a mean of 10.4 days. Post-FDS bleeding was associated with admissions for bowel ischemia (p<0.01) or a prior episode of GI bleeding (p=0.02). 9(36.0%) patients with post-FDS bleeding were on anticoagulants or antiplatelet agents, compared to 99(41.6%) patients without (p=0.59). 17(68.0%) patients with post-FDS bleeding underwent endoscopy; 9(52.9%) had rectal ulcers and 3(17.6%) had rectal ischemia. The mean number of endoscopic procedures to achieve hemostasis was 1.9. The mean length of ICU stay among patients with post-FDS bleeding was 25 days, compared to 16.8 days for those without (p=0.25). 1 patient required surgery for bleeding, 1 sustained a perforation, and 5 required FDS discontinuation due to pain. 3 patients (1.1%) died after post-FDS bleeding.

**Conclusions:**

In our cohort, post-FDS bleeding was seen in a substantial proportion of patients. Post-FDS bleeding was more likely in patients admitted to ICU for prior GI bleeding and ischemic colitis. Post-FDS bleeding resulted in longer ICU stays, though the difference was not statistically significant. Complications from FDS are difficult to predict, and FDS usage in ICUs should be minimized.

Characteristics of patients with and without post-FDS bleeding

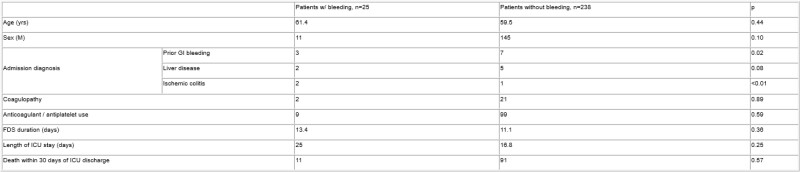

**Funding Agencies:**

